# Tightening policy and housing price bubbles: Examining an episode in the Chinese housing market

**DOI:** 10.1371/journal.pone.0309483

**Published:** 2024-09-06

**Authors:** Kwangwon Ahn, Minhyuk Jeong, Jinu Kim, Domenico Tarzia, Ping Zhang

**Affiliations:** 1 Department of Industrial Engineering and Center for Finance and Technology, Yonsei University, Seoul, Republic of Korea; 2 HSBC Business School, Peking University, Shenzhen, China; 3 GF Fund Management, Guangzhou, China; Damascus University, SYRIAN ARAB REPUBLIC

## Abstract

Housing markets are often characterized by price bubbles, and governments have instituted policies to stabilize them. Under this circumstance, this study addresses the following questions. (1) Does policy tightening change expectations in housing prices, revealing a regime change? (2) If so, what determines the housing market’s reaction to policy tightening? To answer these questions, we examine the effects of policy tightening that occurred in 2016 on the Chinese housing market where a price boom persisted in the post-2000 period. Using a log-periodic power law model and employing a modified multi-population genetic algorithm for parameter estimation, we find that tightening policy in China did not cause a market crash; instead, shifting the Chinese housing market from faster-than-exponential growth to a soft landing. We attribute this regime shift to low sensitivity in the Chinese housing market to global perturbations. Our findings suggest that government policies can help stabilize housing prices and improve market conditions when implemented expediently. Moreover, policymakers should consider preparedness for the possibility of an economic crisis and other social needs (e.g., housing affordability) for overall social welfare when managing housing price bubbles.

## 1. Introduction

Housing prices in China have risen at a rate that is nearly twice as fast as the national income in the past decades [1]. Massive rural–urban migration generated a significant increase in demand for housing in major metropolitan areas as the populations of 35 major metropolitan areas increased by 63.01 million (27.86%) between 1998 and 2015, and per capita living space in metropolitan China increased from 18.7 square meters in 1998 to 36.0 square meters in 2015. This accelerated urbanization has driven housing market boom in China since 2000. Moreover, as employment and disposable income have risen, so has the demand for larger dwellings, which has strengthened the housing market boom [2]. The Chinese housing market has advanced the national economy by contributing to output value and the labor market and has also been subject to policy interventions.

In response to the housing market boom, diverse policies have been suggested; for example, land supply policy [2] and anti-neoliberal policy. Excessive deregulation originating from neoliberal governing strategies have resulted in overbuilding and a real estate boom in China [3]. To mitigate house price increases, the Chinese government tightened rules on home purchases and related borrowing in 2007, 2009, 2013, and 2016. These restrictions have often been linked to households’ residency status (*hukou*) in areas where real estate purchases are intended, requiring local residency or other supporting evidence of long-term residence. To temper speculative demand, higher mortgage rates and reduced loan-to-value (LTV) ratio requirements for investment or second home purchases were also implemented. In addition, the government imposed minimum ownership periods ranging from two to five years before a property could be resold. However, whenever tighter policies placed too much pressure on prices and the stock of unsold housing, the authorities responded by relaxing lending policies and implementing tax exemptions. Furthermore, more direct policies were implemented to support home purchases, such as expanding the scope of shantytown reconstruction and increasing the proportion of monetized resettlement for shantytown reconstruction (A shantytown is a special and age-old urban residential space type that can generally be described as uninhabitable informal housing and areas inhabited by high-density, low-income groups). Historically, regulatory policy has been employed to control real estate booms and busts, effectively curbing house prices [4–7]. A key question naturally follows as to whether government policies—tightening and loosening episodes—have successfully corrected and stabilized the Chinese housing market.

**Fig 1:**
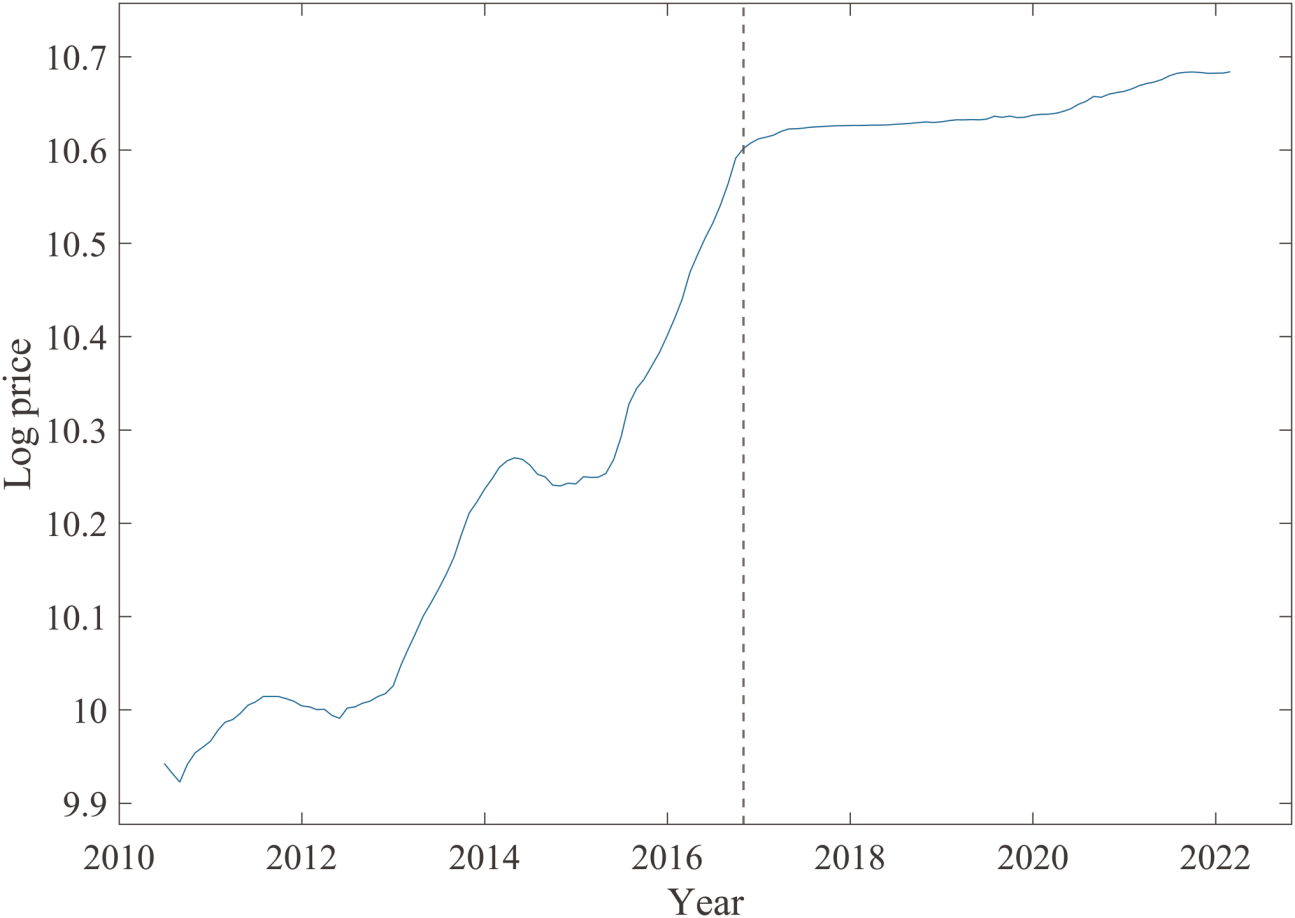
Average house price in China’s first-tier cities (2010–2022). The black dashed line represents the 2016 tightening policy implementation.

Fig 1 presents the average house price in China’s first-tier cities (Beijing, Shanghai, Guangzhou, and Shenzhen) from 2010 to 2022. The black dashed line denotes the most recent episode of housing restriction policies in 2016. This policy must be distinguished from previous policies because property price inflation has persisted, particularly in medium-sized and smaller cities, despite the introduction of a large number of house purchase restrictions and more stringent LTV requirements. Indeed, by 2016, housing price inflation had risen sharply, and authorities had growing concerns about potential bubbles, sharp increases in leveraged property purchases, and related risks to financial stability. In response, local authorities tightened policies again, announcing more measures as housing price inflation persisted. In some areas, required down payments reached 80% of the purchase price. Furthermore, to limit excessive speculation, many cities implemented policies that prohibited new home buyers from reselling within two or three years. However, property prices continued to rise despite the restrictions as shown in Fig 1. The 2016 tightening policy did not trigger a market crash but stopped the sharp increase in housing prices.

This study aims to answer two questions. (1) Does a tightening policy change expectations about housing prices, revealing a regime change? (2) If so, what determines the reaction of housing markets to the tightening policy? These questions are crucial because housing prices can be overvalued and it is of interest for researchers and policymakers to understand how policies for housing price bubbles should be formulated [8]. In addition, considering that a housing bubble was one of the primary reasons for the global financial crisis in 2008 [9], decelerating bubble build-up in housing markets and avoiding a housing price crash, which can trigger an economic crisis, is an urgent concern. We analyze the case of the Chinese housing market in 2016 when prices had been increasing for decades to answer these questions. Specifically, we examine the reaction of the Chinese housing market to the Chinese government’s tightening policy in comparison to similar episodes in Japan and South Korea. We employ the log-periodic power law (LPPL) model to explore the dynamics of China’s housing market. Specifically, we relax the upper constraint on *β*, although the existing literature assumes that the exponent of the power law *β* should be bounded between 0 and 1 [10–12]. We also implement a self-defined multiple population genetic algorithm (MPGA) to fit the nonlinear model.

The findings reveal a speculative bubble prior to policy implementation. When the upper constraint on the power law exponent is relaxed, the Chinese housing market exhibits super-exponential price growth because *β* is still less than one. All other parameters are consistent with the LPPL’s bubble scenario. In contrast, relaxing the constraint allows us to determine what occurred following the implementation of the 2016 tightening policy. After the policy was implemented, the Chinese housing market experienced a regime shift to a soft landing state. The power law exponent is greater than one, indicating a scenario in which housing prices decelerated. We also conduct a postmortem analysis, examining the Tokyo real estate bubble to determine whether loosening the constraint on *β* effects the fit of the LPPL model. When *β* is allowed to be greater than one, LPPL still captures the super-exponential growth of the Japanese housing market. We also provide evidence that the 1987 policy tightening triggered a market crash. Lomb–Scargle spectral analysis [13] and unit root tests confirm the validity of our framework for examining the Chinese housing market and its log-periodic behavior.

We find that the Chinese housing market differs because of its distinctive sensitivity to general global influence, as measured by susceptibility. A lower susceptibility in the Chinese housing market indicates a reduced impact of policy tightening in comparison to the Japanese real estate market, which induced a soft landing rather than a crash. A robustness test based on the South Korean housing market further supports our findings. Overall, we determine that government policies are an appropriate and effective method for market stabilization and improvement when implemented in a timely manner.

This study is related to recent studies applying LPPL and its extensions to bubbles in the real estate market. Although the first influential studies on real estate bubbles date back to the 1980s and 1990s [14–17], relatively few applications of the LPPL model have been conducted on real estate; however, the approach has become popular in recent years. Zhou and Sornette [18] examined the real estate market in the United States, predicting that 22 states exhibited obvious inclinations of a fast growing bubble and the turning point of the bubble would likely occur around mid-2006. Ardila et al. [19] applied LPPL to the Swiss real estate market and revealed evidence of 11 critical districts with signs of speculative bubbles and seven districts with bubbles that had already burst. Ardila et al. [20] proposed a factor model combining LPPL and a large number of macroeconomic variables, demonstrating that the model can forecast the end of bubbles and identify highly relevant variables during a bubble scenario. Ahn et al. [21] investigated whether the LPPL model could predict a bubble crash in the real estate investment trust market, determining that investors’ strong herding behavior and market efficiencies are required (e.g., the bubble crash around the global financial crisis) for the application of the LPPL model. Qun et al. [22] applied a quantile regression calibration approach to determine possible turning points in Beijing, Shanghai, Shenzhen, Guangzhou, Tianjin, and Chengdu, revealing a mixed scenario in which Beijing, Tianjin, and Chengdu were at a high risk of approaching a turning point, while a change of regime was likely to occur for Shanghai and Shenzhen. Zhi et al. [23] conducted a series of diagnostic bubble analyses across 35 representative Chinese cities, finding that 10 out of the 35 cities exhibited positive LPPL signals. Shao et al. [24] applied the LPPL model to the housing market in Wuhan, providing a rough forecast of the critical time but without estimation results; they focused on policy sorting, natural language, and visualization to provide policy recommendations. Jang et al. [25] investigated the South Korean real estate market bubble from the perspective of government intervention in prices, revealing that housing policy effectively moderated the rapid rise of housing prices.

This study differs from Qun et al. [22] and Zhi et al. [23] in two ways. First, we find the 2016 tightening policy to represent a turning point (either a crash or a regime change) and policy implementation marked the end point of super-exponential price growth. Specifically, we evaluate whether the 2016 tightening policy was an efficient means of discouraging short-term speculative investors while preventing a large-scale exit from real estate markets, which would result in a crash. Second, we propose a modified MPGA that combines a two-point and uniform crossover to give each population different characteristics. By applying a migration operator between different populations and a retention operator for elite individuals, we improve the algorithm’s convergence speed and the efficiency of global search. To the best of our knowledge, this is the first attempt to use a modified MPGA combined with relaxing restrictions on the power law exponent in the context of the LPPL model.

The remainder of the paper is structured in the following way. Section 2 explains the theoretical framework of our study’s LPPL model. Section 3 describes the research methodology. Section 4 presents the results of the empirical analysis. Section 5 concludes with final remarks.

## 2. Theoretical framework

We employ the LPPL framework [26] to determine whether housing prices exhibit any sign of unsustainable speculation. This framework is characterized by faster than exponential growth patterns caused by herding behavior and positive feedback mechanisms, coupled with periodic oscillation dynamics caused by heterogeneous interactions between fundamentalists and trend chasers [11, 25].

### 2.1. Price dynamics

The LPPL framework starts by assessing the dynamics of housing price (*p*_*t*_), taking the following form:

$$dp_{t}=u_{t}p_{t}dt-\kappa p_{t}dj,$$ (1)

where *u*_*t*_ is a drift term and *dj* represents a discontinuous jump with the dummy variable *j* that switches to *j* = 0 prior to the critical time and to *j* = 1 afterward. The component size equals *κ* ∈ (0, 1). After we assume rational expectations and no arbitrage condition, the price process would be a martingale:

$$E_{t}\left[dp_{t}\right]=u_{t}p_{t}dt-\kappa p_{t}h_{t}dt=0,$$ (2)

where *h*_*t*_ ≡ *E*[*dj*/*dt*] is the crash hazard rate. We can obtain *u*_*t*_ = *κh*_*t*_, meaning that rational traders accept the risk of a bubble crash if they earn high enough profits to compensate for the risk. Then, the stochastic differential equation of the price dynamics before the crash is the following ordinary differential equation:

$$d\;\text{ln}\;p_{t}=\kappa h_{t}dt,$$ (3)

with the following solution:

$$\text{ln}\;\left(p_{t}/p_{t_{0}}\right)=\kappa \int_{t_{0}}^{t}h_{\tau }d\tau .$$ (4)

### 2.2. Microscopic modeling of the crash

Johansen et al. [26] introduced a microscopic model for an individual trader’s behavior. Specifically, the authors assumed that individual agent *i* can be in one of two states: *s*_*i*_ ∈ {−1, +1}. The authors then postulated that agent *i*’s state is determined as follows:

$$s_{i}=sign\left(K\sum_{j\in N\left(i\right)}s_{j}+\sigma \varepsilon_{i}+G\right),$$ (5)

where *K* is the tendency toward imitation, *N*(*i*) is the set of traders influencing agent *i*’s behavior, *σ* is the tendency toward idiosyncratic behavior, and *G* is a global influence term. *ε*_*i*_ is assumed to be independent and follows the standard normal distribution. The average state of the system is then defined as follows:

$$M\equiv \frac{1}{I}\sum_{i=1}^{I}s_{i},$$ (6)

where *I* is the total number of individual traders in the system. The system’s susceptibility is defined as follows:

$${\chi =\left.\frac{d(E\left[M\right])}{dG}\right|}_{G=0},$$ (7)

which quantifies the sensitivity of the average state of the system to a small global influence [26].

### 2.3. Log-periodic power law model

Johansen et al. [26] proposed a hierarchical diamond lattice structure for the network of traders. The first-order expansion of the general solution is as follows:

$$\chi \approx A_{0}{\left(K_{c}-K\right)}^{-\gamma }+A_{1}{\left(K_{c}-K\right)}^{-\gamma }\text{cos}\;\left[\omega \;\text{ln}\;\left(K_{c}-K\right)+\psi \right],$$ (8)

where *A*_0_, *A*_1_, *ω*, and *ψ* are constants. *γ* is the critical exponent of susceptibility, and *K*_*c*_ is a critical point: the susceptibility is finite when *K* < *K*_*c*_, but goes to infinity as *K* gets closer to *K*_*c*_ (i.e., the value of *K* at the critical time *t*_*c*_).

If we assume that *K* is a function of time *t* and evolves smoothly over time, *K*(*t*) can be approximated using a first-order Taylor expansion as follows:

$$K_{c}-K\left(t\right)\approx \theta \left(t_{c}-t\right),$$ (9)

where *θ* is a constant. Johansen et al. [26] posited that the crash hazard rate behaves the same with the susceptibility near the critical point, and the crash hazard rate approximates to the following:

$$h_{t}\approx {\alpha \left(t_{c}-t\right)}^{\beta -1}\{1+\gamma \;\text{cos}\;[\omega \;\text{ln}\;\left(t_{c}-t\right)+\varphi ]\},$$ (10)

where *t* precedes the critical time *t*_*c*_ (i.e., 0 < *t* < *t*_*c*_). That is, *h*_*t*_ includes a component of power law singularity caused by noisy traders’ self-reinforcing herding behavior, i.e., *α*(*t*_*c*_ − *t*)^*β*−1^, as well as an oscillating component caused by heterogeneous interactions between fundamentalists and trend chasers, i.e., *γ* cos[*ω* log(*t*_*c*_ − *t*) + *φ*]. The power law singularity embodies the positive feedback mechanism that is driven by self-reinforcing beliefs, whereas log-periodicity captures the tug-of-war between the two types of investors leading to large-scale volatility and singularity, or critical time *t*_*c*_ [25, 27].

By using the solution of ordinary differential equation in subsection 2.1 (Eq (4)), we obtain the following:

$$Y_{t}≔\text{ln}\;p_{t}=B_{0}+{B_{1}\left(t_{c}-t\right)}^{\beta }+{C\left(t_{c}-t\right)}^{\beta }\text{cos}\;[\omega \;\text{ln}\;\left(t_{c}-t\right)+\phi ],$$ (11)

where *B*_0_ > 0 is the log price at critical time, i.e., $B_{0}=\text{ln}\;\;p_{t_{c}}$, *B*_1_ < 0 is equal to the increase in log price before the critical time, *C* ∈ (*B*_1_, −*B*_1_) controls the magnitude of oscillations around the exponential trend, and *ϕ* ∈ [0, 2*π*] is a phase parameter.

## 3. Methodology

### 3.1. Bubble detection

Bubbles are typically defined as explosive price growth. In our context, a bubble is defined as an accelerating price that is faster than exponential (super-exponential). The faster than exponential implies that the growth rate is increasing, indicating a positive feedback process that reinforces growth. To detect the presence of a bubble, we distinguish between (standard) exponential and super-exponential growth. The former is characterized by the logarithm of the price being linear in time, as follows:

$$\text{ln}\;p_{t}=a+bt+\varepsilon_{1}.$$ (12)

The most basic extension of Eq (12) that results in super-exponential growth is as follows:

$$\text{ln}\;p_{t}=a+bt+{ct}^{2}+\varepsilon_{2}.$$ (13)

The null hypothesis is *c* = 0. If the price process is found not to follow standard exponential growth, it can then be described as super-exponential growth. The improvement in the goodness of fit resulting from the quadratic term is measured by the root mean square of residuals, which is *RMS*_1,*i*_ for standard exponential growth and *RMS*_2,*i*_ for super-exponential growth in Eqs (12) and (13), respectively, as follows:

$$D_{\;i}=\frac{{{RMS}_{1,i}-RMS}_{2,i}}{{RMS}_{1,i}},$$ (14)

where a higher *D* value indicates a higher likelihood that the null hypothesis will be rejected and the quadratic term qualifying a bubble regime is relevant. As a result, we consider housing prices to be growing at a super-exponential rate if *D*_*i*_ ≥ 25% and *c* > 0 hold. If *p*_*t*_ meets both conditions but *p*_*t*−1_ does not, then time *t* is considered to represent the start of the bubble [28, 29].

### 3.2. Fitting the LPPL parameters

The basic form of the LPPL model presented in Eq (11) requires estimating seven parameters. We set the objective function for the parameter estimation as the squared sum of residuals between the observation and the predicted value of the LPPL model as follows:

$$\sum_{i=1}^{T}{\left\{y_{t}-(A+{B\left(t_{c}-t_{i}\right)}^{\beta }+{C\left(t_{c}-t_{i}\right)}^{\beta }\;\text{cos}\;\left[\omega \;\text{log}\;\left(t_{c}-t_{i}\right)+\phi \right])\right\}}^{2},$$ (15)

where *y*_*t*_ and *T* denote the log of observed price at time *t* and the number of observations in the dataset, respectively.

Let *f*(*t*; *β*, *t*_*c*_) = (*t*_*c*_ − *t*)^*β*^ and *g*(*t*; *β*, *ω*, *t*_*c*_, *ϕ*) = (*t*_*c*_ − *t*)^*β*^ cos[*ω* log(*t*_*c*_ − *t*) + *ϕ*], and we then obtain the following optimization problem:

$$\underset{\beta ,\omega ,t_{c},\phi }{\text{min}}\;\sum_{i=1}^{T}{\left\{y_{t}-\left(A+Bf\left(t_{i};\beta ,t_{c}\right)+Cg\left(t_{i};\beta ,\omega ,t_{c},\phi \right)\right)\right\}}^{2},$$ (16)

where the coefficients *A*, *B*, and *C* can be estimated using the ordinary least squares method given the four parameters *β*, *ω*, *t*_*c*_, and *ϕ*.

Previous studies have reported that the conditions for a bubble regime include 0 < *β* < 1 and *B* < 0 [10, 11, 25, 27, 30]. Specifically, *β* < 1 ensures that the expected log price diverges at the critical time and *β* > 0 ensures that the log price remains finite at *t*_*c*_. The two conditions ensure super-exponential growth toward the critical time (*t*_*c*_), implying that the crash hazard rate accelerates over time. To examine the regime shift before and after policy implementation, we relax the upper constraint on *β*; that is, *β* < 1. For *ω*, if the log-periodic oscillations are too fast (large *ω*), then the model would overfit the random noises of the data. In contrast, if the log-periodic oscillations are too slow (small *ω*), they contribute to the trend too much, neglecting the real oscillations in this false trend. However, previous research has demonstrated that log-periodic oscillations are neither too fast nor too slow for financial crashes, e.g., *ω* = 6.36 ± 1.56 [31], and *ω* has been found around the boundaries. Therefore, we only restrict *ω* to be positive as *ω* > 0 is simply the frequency of the fluctuations during the bubble formation [11].

We estimate the four nonlinear parameters (*β*, *ω*, *t*_*c*_, and *ϕ*) using the MPGA. The genetic algorithm (GA) references concepts from biology such as crossover and mutation when searching for optimal parameters. However, unlike the traditional GA, the MPGA generates multiple populations and each population is muted into hundreds of chromosomes, each of which represents a feasible solution for the four nonlinear parameters in the LPPL model. If the optimization criteria are not met, the algorithm creates new populations and restarts the search. As a result, the algorithm enhances prediction accuracy and minimizes the impact of other factors such as crossover and mutation probability. Moreover, the MPGA avoids the local-trap and premature convergence problems of the standard GAs [32]. The specific MPGA procedure to optimize nonlinear parameters is presented in S1 Appendix. In particular, we employ a modified MPGA to determine the optimal parameter values in the LPPL model. We first partition the sample space into subintervals to mitigate the impact of a specific sample interval and then use the MPGA to fit the LPPL model within each subinterval.

### 3.3. Lomb–Scargle spectral analysis and unit root tests

We test log-periodicity using Lomb–Scargle spectral analysis [13] to detect periodic oscillation in our irregularly sampled data. The Lomb–Scargle analysis returns a series of frequencies (*ω*) and associated periodograms (*P*(*ω*)) that represent the power of the frequencies. The frequency with the maximum power is taken as the least squares frequency (*ω*_*Lomb*_). We perform the Lomb–Scargle analysis on detrended residuals as follows:

$$r\left(t\right)={\left(t_{c}-t\right)}^{-\beta }\left[\text{ln}\;p_{t}-A-B{\left(t_{c}-t\right)}^{\beta }\right].$$ (17)

Specifically, we estimate the power at frequency *ω* as follows:

$$P\left(\omega \right)=\frac{1}{2}\left(\frac{{\left[\sum_{i}r_{s}\left(t\right)\text{cos}\;\omega \left(\nu_{i}-\tau \right)\right]}^{2}}{\sum_{i}{\text{cos}}^{2}\;\omega \left(\nu_{i}-\tau \right)}+\frac{{\left[\sum_{i}r_{s}\left(t\right)\text{sin}\;\omega \left(\nu_{i}-\tau \right)\right]}^{2}}{\sum_{i}{\text{sin}}^{2}\;\omega \left(\nu_{i}-\tau \right)}\right),$$ (18)

where *r*_*s*_(*t*) is the standardized *r*(*t*) and *ν*_*i*_ is the log of (*t*_*c*_ − *t*) for observation *i*; that is, ln(*t*_*c*_ − *t*). *τ* is the time delay which satisfies the following:

$$\text{tan}\;2\omega \tau =\frac{\sum_{i}\;\text{sin}\;2\omega \nu_{i}}{\sum_{i}\;\text{cos}\;2\omega \nu_{i}}.$$ (19)

In the LPPL framework, the log-periodic oscillations result from the cosine part in Eq (11). As a result, if no log-periodicity is evident in the residuals, the angular frequency (*ω*_*Lomb*_) should be close to *ω*_*LPPL*_. The two frequencies are considered to be close if the absolute value of their difference is less than 0.3 [33]. Moreover, the *p*-value for the null hypothesis that the data is nonperiodic at a given frequency *ω* is calculated as 1 − [1 − exp(−*P*(*ω*))]^*M*^, where *M* is the number of test frequencies. *M* is determined as the number of independent frequencies which are approximated as −6.362 + 1.193*T* + 0.00098*T*^2^ [34].

Furthermore, if the logarithmic price in the bubble regime is attributed to a deterministic LPPL component, a mean-reversal Ornstein–Uhlenbeck (OU) process can be used to model residuals [12]. The test for the OU property of residuals can be translated into an AR(1) test for the corresponding residuals. Therefore, we conduct unit root tests on the LPPL residuals employing augmented Dickey–Fuller (ADF) and Phillips–Perron (PP) tests. Rejecting the null hypothesis suggests that residuals are stationary and compatible with the OU process in the residuals. The optimal lag for the ADF test is determined by the minimum Akaike information criterion, while the lag for the PP test is calculated as 12(*n*/100)^1/4^ [35].

## 4. Empirical analysis

### 4.1 Risk of bubble crash

We use monthly data on the average house prices in China’s first-tier cities from June 2010 to February 2022, which are obtained from the WIND database. Table 1 presents the summary statistics of our data. All data are annualized log returns. The mean and standard deviation decreases during the postimplementation period, indicating decelerated price growth and reduced volatility. Moreover, both skewness and kurtosis increase significantly, suggesting a change in distribution following policy implementation. We conjecture that the summary statistics suggest a plausible link between policy implementation and the housing market.

**Table 1:** Descriptive statistics.

	Whole period	Pre-regulation	Post-regulation
	(July 2010–February 2022)	(July 2010–October 2016)	(October 2016–February 2022)
N	140	76	64
Mean	0.064	0.104	0.015
SD	0.100	0.121	0.019
Max.	0.419	0.419	0.071
Min.	−0.123	−0.123	−0.019
Skew.	1.070	0.147	0.872
Kurt.	0.842	−0.598	0.430

Note: This table presents the mean (Mean), standard deviation (SD), maximum (Max.), minimum (Min.), skewness (Skew.), and excess kurtosis (Kurt.) for log returns of the Chinese housing market for the entire period and the periods before (Pre-regulation) and after (Post-regulation) tightening policy implementation.

**Fig 2:**
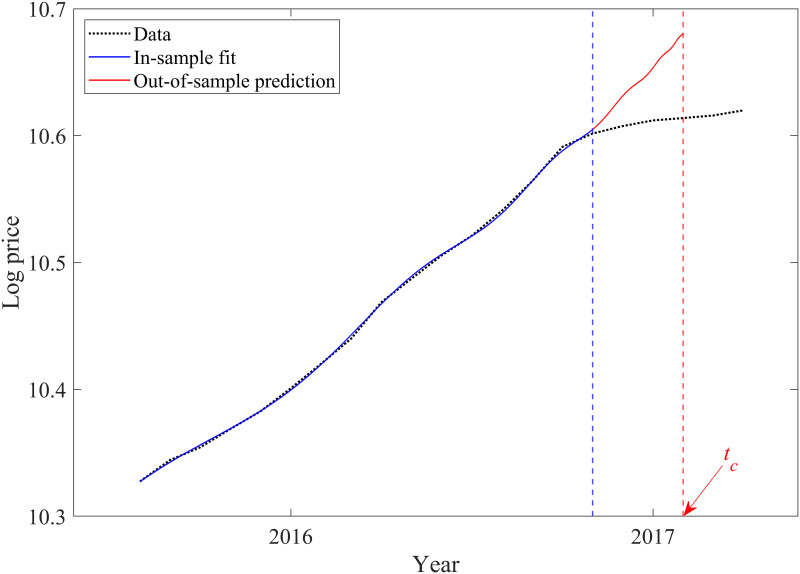
Log-periodic power law fit to the Chinese housing market bubble preceding the 2016 policy tightening. The blue and red solid lines indicate in-sample fit and out-of-sample predictions, respectively. The black dotted line represents the housing price in China. The blue dashed line indicates the 2016 tightening policy implementation. The red dashed line indicates the critical time predicted by the log-periodic power law model.

As noted previously, the tightened regulations appear to be effective in reducing volatility in the housing market. We use the LPPL model to examine the effectiveness of government policies. Using a 12-month moving average, we identify the start of the bubble in July 2015 as *D*_*July*2015_ = 0.3819 and *c*_*July*2015_ = 0.0696, while *D*_*June*2015_ = 0.0947 and *c*_*July*2015_ = 0.0457. The blue solid line in Fig 2 is an in-sample estimate from the beginning of the bubble, while the blue dashed line identifies the time of tightening policy implementation. Comparing data and model responses, we conclude that our model fits very well for the Chinese housing market.

**Fig 3:**
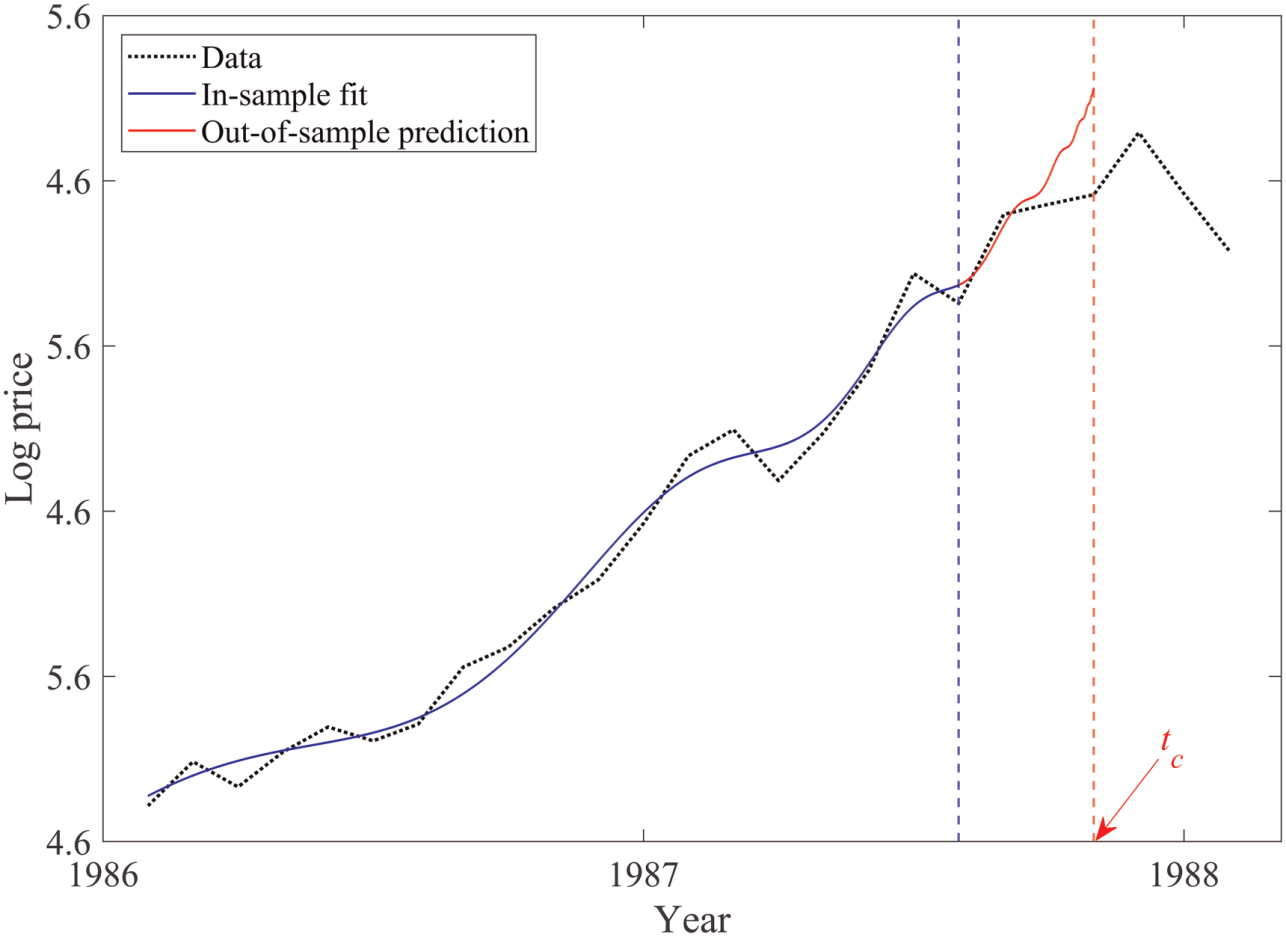
Log-periodic power law fit to the Japanese housing market bubble preceding the 1987 tightening policy. The blue and red solid lines indicate in-sample fit and out-of-sample predictions, respectively. The black dotted line represents the house price in Japan. The blue dashed line indicates the 1987 tightening policy implementation. The red dashed line indicates the critical time predicted by the log-periodic power law model.

We then conduct a postmortem analysis of the late 1980s Tokyo real estate bubble to determine whether easing the constraints on *β* affects the model’s detection power. We choose the Tokyo real estate bubble because it bears a striking resemblance to the current Chinese real estate market. We assume that the Japanese real estate bubble began in January 1986 because *D*_*January*1986_ = 0.3971 and *c*_*January*1986_ = 0.3236, while *D*_*December*1985_ = 0.2156 and *c*_*December*1985_ = 0.2154. Fig 3 presents an in-sample estimate from the start of the bubble to the policy tightening that was implemented in July 1987. Comparing data and LPPL model responses, we conclude that our model also fits well for the Japanese housing market.

Table 2 summarizes the parameter estimates. For both housing markets, two parameters confirm a faster than exponential acceleration of log prices (i.e., *B* < 0 and 0.1 < *β* < 0.9) [12]. Consistent with previous studies [12, 31], the *ω* values are 8.92 and 7.57, respectively.

**Table 2:** The best fit of log-periodic power law parameters.

Panel A: Chinese Housing Market								
End time	A	B	C	*β*	*t*_*C*_	*ω*	*ϕ*	RMSE
10/2016	10.69	–0.2611	–0.0073	0.8651	02/12/2017	8.92	5.45	0.17%
Panel B: Japanese Housing Market								
End time	A	B	C	*β*	*t*_*C*_	*ω*	*ϕ*	RMSE
07/1987	5.22	–0.6217	–0.0404	0.7032	10/31/1987	7.57	5.17	2.26%

Note: This table presents the parameter estimates of the log-periodic power law model for the housing markets in China and Japan. The root-mean squared error (RMSE) is reported in the last column. End time references the month of policy implementation. Panel A reports the parameter estimation for the Chinese housing market. Panel B reports the parameter estimation for the Japanese housing market.

### 4.2 Policy impact

We then conduct out-of-sample forecasting for the post-implementation period, which is after October 2016 and July 1987 for China and Japan, respectively. Although the LPPL model indicates a critical time in the first half of 2017, market prices did not rapidly increase without the collapse of the bubble. This implies that the government policies that were implemented to curb a boom in the housing market were accurately timed. In contrast, after reaching its peak, Tokyo’s housing price index began to fall abruptly in November 1987, which is consistent with the critical time in Table 2. This suggests that the Bank of Japan’s tightened monetary policy caused a sharp decline in the growth of money supply, which triggered a housing market crash.

Despite similar credit loan restrictions, Figs 2 and 3 reveal different reactions from the Japanese and Chinese housing markets following policy implementation. We next investigate how LPPL parameters changed near the critical time, changing the end date of the in-sample estimation for the two housing markets to examine the sensitivity of the LPPL parameters.

Table 3 Panel A presents the MPGA estimates for the Chinese housing market. End time spans from two months prior to policy implementation to 11 months following implementation, from August 2016 to September 2017, to include information leakage and policy transmission near the implementation. Initially, prices exhibited upward acceleration with the coefficient *B* lower than zero. At the same time, the coefficient *β* < 1 reveals a faster than exponential acceleration of log prices until October 2016, supporting the argument that a housing bubble is evident and could burst soon. Prior to the 2016 tightening policy, the value of *ω* is between 8.92 and 12.91, which is consistent with existing literature [12, 31]. However, following policy implementation, the market abruptly changed and *β* became higher than one, rising from 0.8651 to 1.7375, indicating a reduced crash hazard rate. Parameter *ω* suddenly dropped to 0.86 in November 2016, implying the beginning of a new regime that is representative of a soft landing scenario. The empirical results validate the argument that government policies enacted to curb a housing market boom were accurately timed and the housing market transitioned to a soft landing state.

**Table 3:** The best fit of log-periodic power law parameters near policy implementation.

Panel A: Chinese Housing Market								
End time	*A*	*B*	*C*	*β*	*t*_*C*_	*ω*	*ϕ*	RMSE
08/2016	11.00	−0.4594	0.0050	0.5710	07/23/2017	12.91	3.59	0.12%
09/2016	10.74	−0.2928	−0.0064	0.7721	03/01/2017	9.02	4.95	0.12%
10/2016	10.69	−0.2611	−0.0073	0.8651	02/12/2017	8.92	5.45	0.17%
11/2016	10.61	–0.2010	0.1995	1.7375	12/01/2016	0.86	4.63	0.35%
12/2016	10.61	−0.1618	−0.1618	1.7871	12/31/2016	1.02	1.28	0.34%
01/2017	10.61	−0.1113	0.0802	1.2727	02/02/2017	1.54	3.26	0.34%
02/2017	10.62	−0.1008	−0.0747	1.2712	02/28/2017	1.73	6.14	0.33%
03/2017	10.62	−0.1016	0.0609	1.3124	03/31/2017	2.04	2.78	0.33%
04/2017	10.62	−0.0967	0.0515	1.3930	04/30/2017	2.34	2.57	0.33%
05/2017	10.62	−0.0856	−0.0413	1.6421	05/31/2017	2.69	5.63	0.33%
06/2017	10.62	−0.0744	0.0258	2.1724	06/30/2017	3.45	2.58	0.34%
07/2017	10.62	−0.0626	0.0206	2.3656	07/31/2017	3.85	2.30	0.34%
08/2017	10.63	−0.0513	0.0168	2.5438	08/31/2017	4.17	1.98	0.33%
09/2017	10.63	−0.0412	−0.0136	2.7275	09/30/2017	4.48	4.78	0.33%
Panel B: Japanese Housing Market								
End time	*A*	*B*	*C*	*β*	*t*_*C*_	*ω*	*ϕ*	RMSE
05/1987	5.22	−0.4191	0.0541	1.0563	08/04/1987	5.36	3.78	2.04%
06/1987	5.46	−0.6419	0.0769	0.3797	07/07/1987	3.26	2.96	2.00%
07/1987	5.52	−0.6217	−0.0404	0.7032	10/31/1987	7.57	5.17	2.26%
08/1987	5.51	−0.6297	0.0409	0.6837	10/21/1987	7.27	2.20	2.21%
09/1987	5.51	−0.6071	−0.0409	0.7344	11/08/1987	7.95	5.01	2.24%
10/1987	5.44	−0.5281	−0.0434	0.8693	11/08/1987	8.09	5.06	2.48%
11/1987	5.55	−0.5697	0.0369	0.8187	01/02/1988	9.51	0.44	2.54%
12/1987	5.47	−0.4001	−0.3337	0.2425	03/27/1988	1.42	4.67	3.20%
01/1988	5.47	−0.4274	0.3694	0.1648	04/13/1988	1.44	1.38	3.13%
02/1988	5.46	−0.3541	0.2872	0.3605	03/07/1988	1.44	1.76	3.08%
03/1988	6.08	−0.9915	−0.2168	0.2121	05/15/1988	2.06	4.32	3.16%
04/1988	10.11	−5.0020	−0.2023	0.0547	07/17/1988	2.54	3.75	3.16%
05/1988	10.35	−5.1400	−0.1846	0.0656	10/29/1988	3.33	2.89	3.29%
06/1988	12.41	−7.0545	−0.1810	0.0593	04/17/1989	4.40	1.26	3.41%

Note: This table presents the parameter estimates of the log-periodic power law models for the housing markets in China and Japan with 14 different end times of input samples spanning near the time of policy implementation. The root-mean squared error (RMSE) is reported in the last column of each panel. Panel A reports the parameter estimation for the Chinese housing market, and Panel B reports the parameter estimation for the Japanese housing market.

Panel B in Table 3 presents the MPGA estimates for the Japanese housing market. Apart from May 1987, all *β*s are lower than one, indicating an increasing crash hazard rate. A negative coefficient *B* and *β* below one, indicating upward acceleration, provide evidence that a bubble was developing and a crash was looming. All the warning signals reveal that a crash in less than three months was imminent. Specifically, *t*_*c*_ − *t* became significantly smaller after July 1987 and its average dropped from 2.58 months to 1.09 months after policy implementation. The critical time was primarily concentrated on late October and early November, which is approximately one month preceding the real crash.

### 4.3 Diagnostic tests

**Fig 4:**
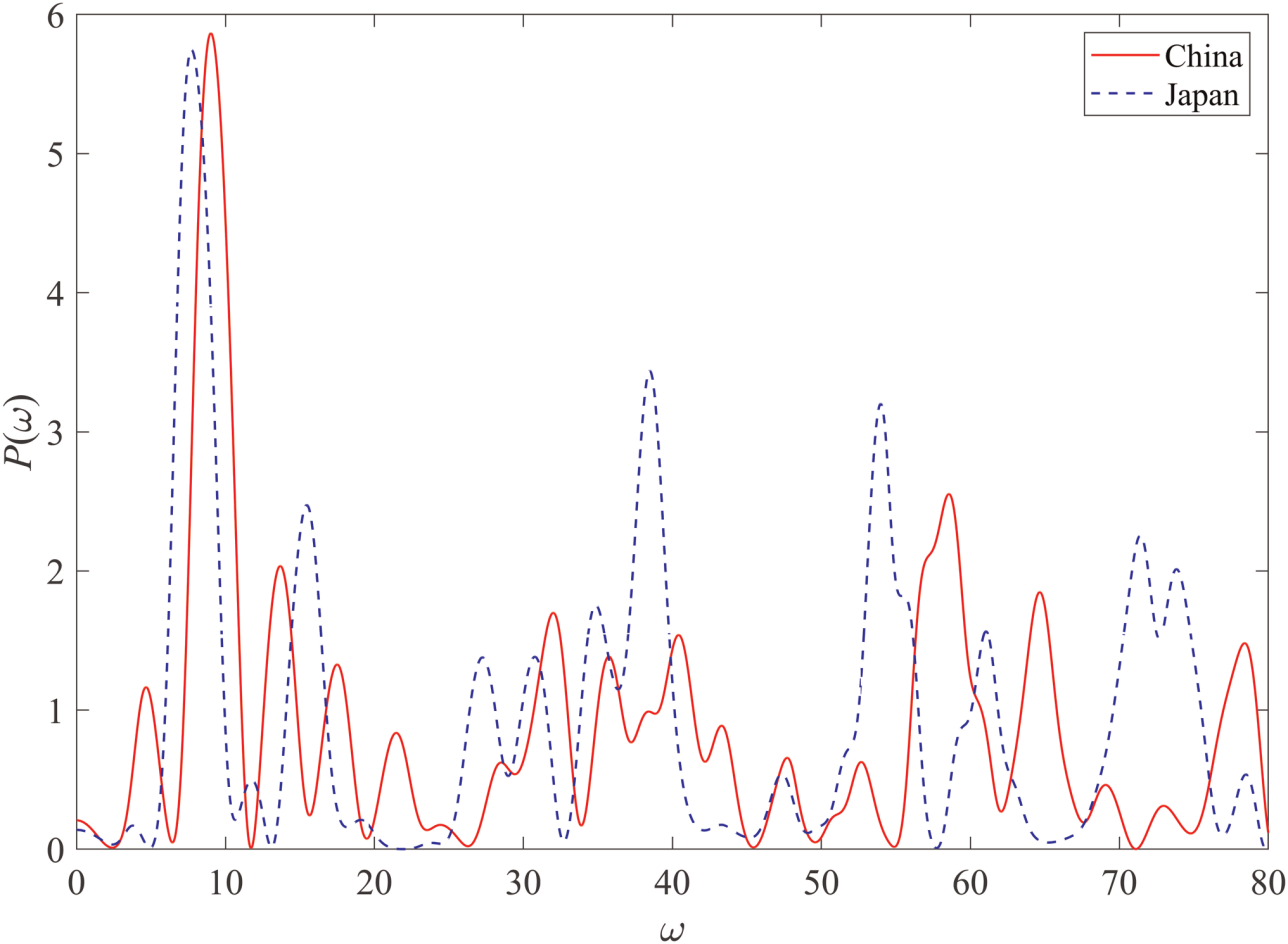
Lomb-Scargle periodograms for the Chinese and the Japanese housing markets. The red solid line indicates the power of the house price in China at each frequency. The blue dashed line indicates that of the house price in Japan.

The Lomb–Scargle periodograms of the detrended residuals are presented in Fig 4. Each point in the Lomb–Scargle periodogram represents the highest peak and its associated angular log-frequency for a given detrended residual series. The red solid line presents the results for the Chinese housing market, and the blue dashed line indicates those for the Japanese housing market. The angular log-periodic frequencies associated with the highest Lomb peaks are 9.02 and 7.72, respectively. The *p*-values of Lomb–Scargle periodograms for Chinese and Japanese housing markets are 0.0362 and 0.0520, respectively. These angular frequencies are close to the values obtained with the parameter estimates that are summarized in Table 2.

Panel A of Table 4 presents the result of unit root tests for which the null hypothesis is that the time series has a unit root and is nonstationary for the Chinese housing market (We also test for homoscedasticity of the LPPL residuals since the ADF test requires that data be homoscedastic, failing to reject the null hypothesis of no heteroskedasticity in the LPPL residual series for Chinese and Japanese housing markets). According to the ADF test, the null hypothesis is rejected for all the series of residuals other than the four series with end times in September 2016, March 2017, May 2017, and August 2017. With the exception of November 2016, the null hypothesis is rejected for all the series of residuals according to the PP test. These findings collectively suggest that the LPPL residuals in the Chinese housing market do not have a unit root and are stationary. Panel B of Table 4 presents the results of unit root tests for the Japanese housing market. According to the ADF test, all the residual series except that with end times in June 1987 reject the null hypothesis. In addition, all the series reject the null hypothesis according to the PP test, indicating that the LPPL residuals in the Japanese housing market also do not have a unit root and exhibit stationarity. Therefore, we conclude that the LPPL residuals in both the Chinese and Japanese housing markets follow a mean-reverting OU process, revealing a key property of the LPPL model [11].

**Table 4:** Unit root tests.

Panel A: Chinese Housing Market						
End time	ADF			PP		
	lags	*t*-stat	*p*-value	lags	*t*-stat	*p*-value
08/2016	3	−4.6159	0.0001	8	−15.4153	0.0000
09/2016	5	0.2837	0.9766	8	−12.9350	0.0000
10/2016	0	−5.2805	0.0000	8	−6.3153	0.0000
11/2016	2	−3.8009	0.0029	8	−2.3900	0.1446
12/2016	1	−3.7244	0.0038	8	−2.7422	0.0670
01/2017	7	−6.2899	0.0000	8	−2.6797	0.0776
02/2017	7	−3.1542	0.0228	9	−2.8695	0.0490
03/2017	8	0.9815	0.9941	9	−2.9233	0.0427
04/2017	6	−2.6656	0.0802	9	−2.9822	0.0366
05/2017	9	−2.3796	0.1476	9	−2.9793	0.0369
06/2017	9	−2.9431	0.0406	9	−2.7515	0.0655
07/2017	9	−2.5775	0.0977	9	−2.7211	0.0705
08/2017	9	−2.4900	0.1179	9	−2.2706	0.0705
09/2017	6	−2.9292	0.0421	9	−2.7209	0.0705
Panel B: Japanese Housing Market						
End time	ADF			PP		
	lags	*t*-stat	*p*-value	lags	*t*-stat	*p*-value
05/1987	6	−2.9960	0.0353	8	−7.4458	0.0000
06/1987	6	−2.2965	0.1731	8	−8.6469	0.0000
07/1987	2	−4.7357	0.0001	8	−7.0930	0.0000
08/1987	2	−4.7831	0.0001	9	−7.3036	0.0000
09/1987	2	−4.9034	0.0000	9	−8.3639	0.0000
10/1987	0	−4.4496	0.0002	9	−5.8982	0.0000
11/1987	0	−5.1667	0.0000	9	−7.3325	0.0000
12/1987	0	−4.3247	0.0004	9	−5.4269	0.0000
01/1988	0	−4.4459	0.0002	9	−5.6060	0.0000
02/1988	0	−4.5246	0.0002	9	−5.6934	0.0000
03/1988	0	−4.7535	0.0001	9	−6.7093	0.0000
04/1988	0	−4.6956	0.0001	9	−6.7401	0.0000
05/1988	0	−4.6630	0.0001	9	−6.9196	0.0000
06/1988	6	−4–2801	0.0005	9	−6.1351	0.0000

Note: This table presents the results of augmented Dickey–Fuller (ADF) and Phillips−Perron (PP) tests for the Chinese housing market (Panel A) and the Japanese housing market (Panel B).

**Fig 5:**
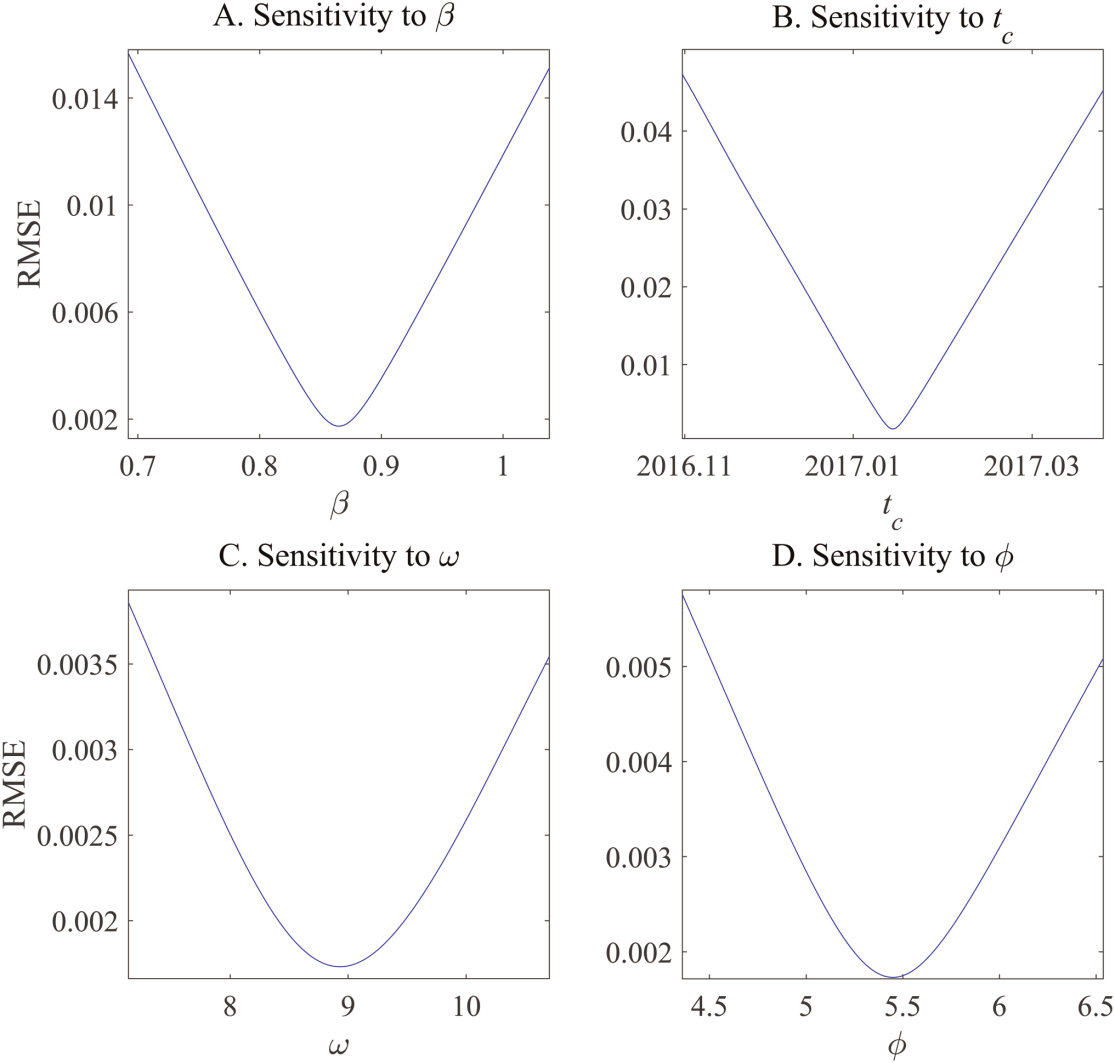
Sensitivity analysis of root mean square errors to the parameters of the log-periodic power law model for the Chinese housing market. Figures A–D represent RMSE sensitivity to *β*, *t*_*c*_, *ω*, and *ϕ*, respectively.

**Fig 6:**
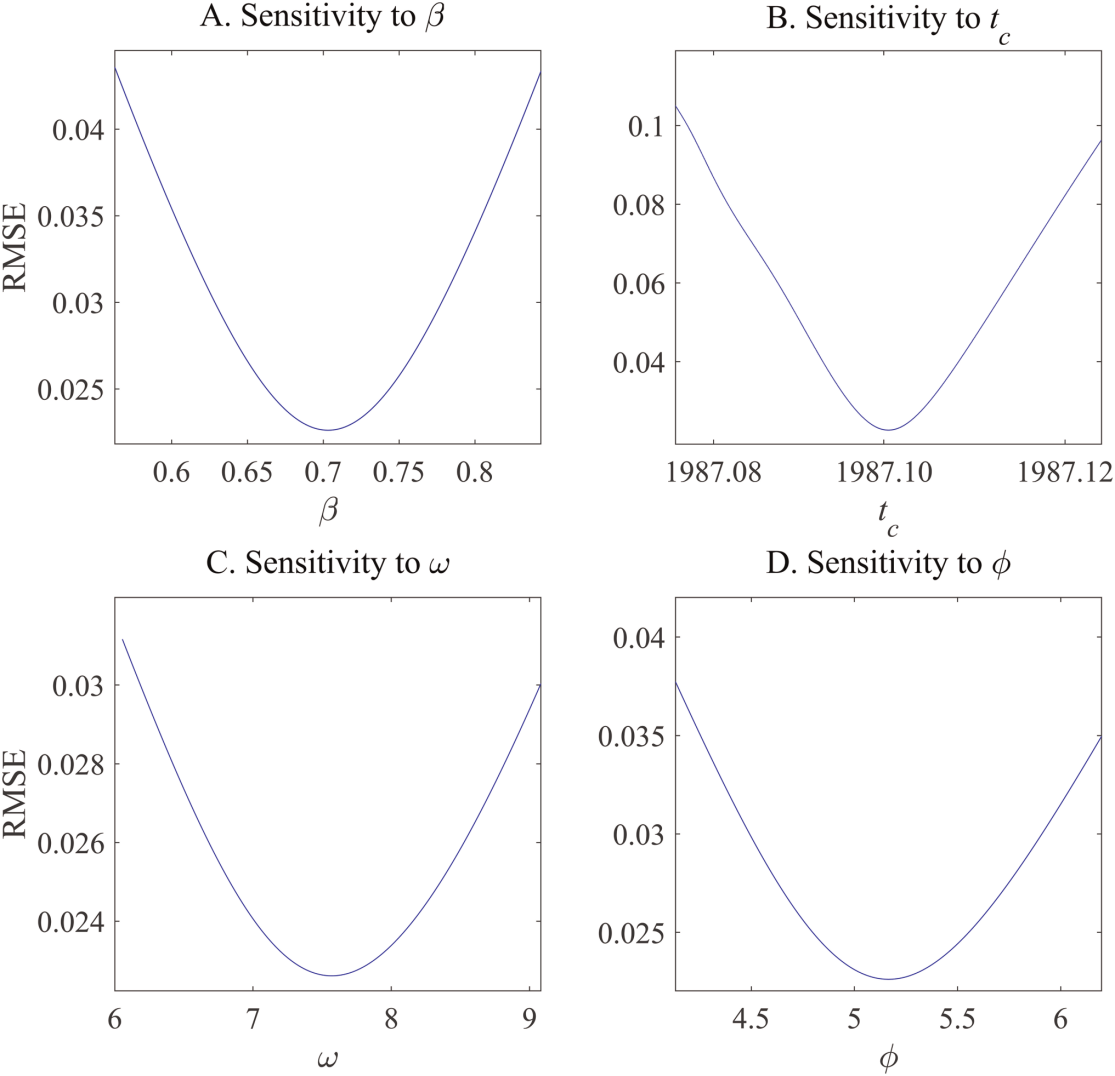
Sensitivity analysis of root mean square errors to the parameters of the log-periodic power law model for the Japanese housing market. Figures A–D represent RMSE sensitivity to *β*, *t*_*c*_, *ω*, and *ϕ*, respectively.

Identifying a bubble using the LPPL model depends on two critical parameters such as *β* and *ω*; therefore, it is crucial to examine the sensitivity of the root mean square errors (RMSEs) of the fitted models to variations in these parameters. Fig 5 illustrates the sensitivity of the LPPL fit reported in Table 2 to variations in each of the four parameters (*β*, *ω*, *ϕ*, and *t*_*c*_, in the case of China), revealing that the RMSE of the fitted model for China is not sensitive to small fluctuations in the chosen values for *β*, *ω*, *ϕ*, and *t*_*c*_. We also conduct sensitivity analysis on the fitted model for the Japanese housing market. As shown in Fig 6, the fitted model for Japan is not sensitive to *β*, *ω*, *ϕ*, and *t*_*c*_ as well, indicating that the model for Japan is also not sensitive to small fluctuations in the four parameters.

### 4.4 Susceptibility

The next question we address is how to explain the different behavior of the Chinese and Japanese housing markets, given the similar credit loan restrictions. Specifically, we want to know what determines a market crash rather than a soft landing. Eq (2) indicates that the higher the probability of a crash is, the faster the price should grow to compensate investors for the increased risk of a crash in the market. In other words, increases in *u*_*t*_ are directly linked to increases in the crash hazard rate (*h*_*t*_), which is strictly related to a system’s susceptibility. Susceptibility measures the ability of agents in a system to reach agreement (i.e., the probability that a large number of agents will take the same position simultaneously) and explains how the emergence of global synchronization from local imitation might cause a crash. The susceptibility of a market *χ* can be estimated by *κh*_*-*_, which can be determined from the estimated LPPL parameters as follows:

$$\kappa h_{t}\approx \frac{\partial Y_{t}}{\partial t}=-B\beta {\left(t_{c}-t\right)}^{\beta -1}-C{\left(t_{c}-t\right)}^{\beta -1}\left[\beta \;\text{cos}\;\left[\omega \;ln\;\left(t_{c}-t\right)+\phi \right]+\omega \;\text{sin}\;\left[\omega \;ln\;\left(t_{c}-t\right)+\phi \right]\right],$$

where the estimated *κh*_*t*_ can be negative, although, by definition, *κ* ∈ (0, 1) and the hazard rate should be positive. We approximate *h*_*t*_ in the LPPL model referencing Sornette [36].

A higher drift in a market in which bubbles are forming indicates that a system is extremely sensitive to even small global perturbation. In such cases, when the time approaches *t*_*c*_, the crash hazard rate (i.e., the probability that a large number of agents will assume the same sell position simultaneously) rises, resulting in an imbalanced market unless prices decrease significantly, at which time prices explode and the probability of a smooth landing plummets to zero.

Therefore, we compare the market susceptibility of the housing markets of China, Japan, and South Korea. We include the South Korean housing market in this analysis for the following reason. Jang et al. [25] examined the real estate market in South Korea, providing evidence that the 2016 South Korean tightening policy was an effective tool for stabilizing housing prices and improving market conditions. However, the subsequent fiscal expansion implemented in November 2017, which was specifically aimed toward economically vulnerable groups—new employees, college graduates, newlyweds, senior citizens, and low-income earners—had no significant impact on the housing market.

Table 5 presents the estimated *κh*_*t*_ to measure market susceptibility, around time *T* (i.e., July 1987 for Japan, October 2016 for China, and October 2016 for South Korea). Different rates in Table 5 explain different reactions to tightening policy, revealing that *κh*_*t*_ is higher in Japan than China and South Korea, which indicates that the Japanese housing market is more vulnerable. The 1987 tightening policy shifted demand to the left, triggering a crash, because the Japanese housing market was extremely sensitive to global perturbations. In contrast, Chinese and Korean housing markets have a lower *κh*_*t*_, indicating lower susceptibility when the tightening policy was implemented. Both markets were less sensitive to minor global fluctuations, the tendency to imitate was weaker, and no strong dominance of one state or position was evident among the agents. As a result, both markets switched to a soft landing state with a *β* greater than one. Furthermore, the low hazard rates and susceptibility help explain why the Korean market did not react to the 2017 fiscal expansion, as documented by Jang et al. [25].

**Table 5:** Approximated hazard rate.

End time	$\kappa^{CHN}h_{t}^{CHN}$	$\kappa^{JPN}h_{t}^{JPN}$	$\kappa^{KOR}h_{t}^{KOR}$
*T* − 2	0.3308	0.5686	0.0427
*T* − 1	0.2835	3.2949	0.0452
*T*	0.2365	0.2985	0.0266
*T* + 1	0.0371	1.0756	−0.0234
*T* + 2	0.0011	1.0172	0.0084
*T* + 3	0.0686	0.8347	0.0526
*T* + 4	0.0147	1.0029	0.0658
*T* + 5	0.0010	−0.5773	−0.0105
*T* + 6	0.0018	−1.4579	0.0197
*T* + 7	0.0001	5.6756	0.0172
*T* + 8	0.0000	1.1341	−0.0063
*T* + 9	0.0000	1.5423	−0.0025
*T* + 10	0.0000	0.8289	−0.0059
*T* + 11	0.0000	0.2629	0.0279
Mean	0.0697	1.1072	0.0184
Median	0.0015	0.9188	0.0185

Note: This table presents the approximated hazard rate (*κh*_*t*_) for Chinese (CHN), Japanese (JPN), and South Korean (KOR) housing markets. *T* corresponds to October 2016 for China, July 1987 for Japan, and October 2016 for South Korea.

In conclusion, our evidence indicates that demand-side policy should be implemented expediently when the system is not vulnerable to small global perturbations and there is no strong agreement among agents to be effective. Conversely, late implementation timing may exacerbate agents’ tendency to imitate others, increase the dominance of one position, and ultimately trigger a market crash.

### 4.5. Discussion on housing policies

We have shown that housing policies should be implemented with accurate timing with low (but not too low) susceptibility to facilitate a soft landing of the housing market bubble. However, the question remains: Is a soft landing after a housing bubble always good? Moreover, we should note that the Chinese housing market is not only influenced by investors’ speculative motives, which was the key concern of the 2016 tightening policy, but also by many other factors, including political ideology, social phenomena, geography, and others. Accordingly, in this subsection, we introduce several notable points when examining housing policies, especially in China.

The Chinese economy is transitioning to a market economy. Moreover, China is distinct from other transitional economies (e.g., Eastern European countries) in that it has only adopted the economic aspects of the market economy, in contrast to most other economies in transition that are fully transitioning to a path of Western liberalism [37]. Hence, we compare the housing market reaction to regulations in China and those in Eastern Europe. After liberalization, Eastern European countries experienced housing booms and busts in the past several decades, with macroprudential policies implemented, some of which have affected housing prices significantly, while others have not [38]. Such boom–bust cycles also occur in developed economies, significantly affected by monetary policies (e.g., interest rates and global liquidity) [39]. Consequently, the housing market fluctuations in Eastern Europe resemble those of mature market economies as, no matter whether they occur in developing or developed economies, housing markets in liberal countries experience bubbles and crashes following the implementation of macroeconomic or financial policies. In contrast, China has sought to avoid systemic risks originating from real estate boom–bust cycles to avoid a crash. As a socialist market economy, the Chinese economy could have successfully relieved the speculative mechanism in the housing market by imposing prudential requirements, monitoring real estate markets, and related approaches [40]. Likewise, the 2016 tightening policy that we focused on this study could also be accurately timed and effective due to state monitoring and market control. In summary, the Chinese housing market reaction to regulations differs from other economies in transition because of differing paths toward market economy development, and the continuing leadership of the Chinese Communist Party could have a significant influence on housing market stability that has been adequate enough to prevent housing prices from experiencing a hard landing.

We can cite cases of housing market soft landings from the history of other transitional countries. For example, the Czech Republic introduced or changed macroprudential policy instruments including the LTV, debt-service-to-income, and debt-to-income limits to reduce risks in the real estate market and mortgage financing from 2015 to 2022 [41]. These demand-side regulations resulted in a significant decrease in the number of new mortgage agreements, cooling down the Czech housing market without a price plummet [42]. Moreover, Vandenbussche et al. [38] found that changes in the minimum capital adequacy ratio (CAR) and marginal reserve requirements (MRRs) related to credit growth significantly decreased housing price inflation in countries in Central, Eastern, and Southeastern Europe (CESEE) during their sample period, which varies by country, ranging from the first quarter of 1997 to the first quarter of 2011. In particular, most of the countries had not experienced a housing price hard landing, indicating that the changes in the minimum CAR or MRRs related to credit growth helped the housing markets in CESEE countries to soft land following sharp price increases. These examples from other regions with transitioning economies again imply the importance of accurately timed policy implementation to prevent the hard landing of housing prices, which we demonstrate in the case of China’s 2016 tightening policy.

Meanwhile, concerns have been expressed that the efforts to cool down demand for properties such as China’s 2016 tightening policy only decrease the number of transactions, rather than property prices [42], resulting in a continuous increase in housing prices. Housing prices surge during a bubble build-up period, and can sometimes be decelerated by effective policy implementation, which we call a soft landing. The problem is that persistent price increases lead to a housing affordability issue wherein people cannot afford to buy a house because of overly high housing prices, excess demand, or scarce supply. Various efforts to alleviate housing unaffordability include supply-side intervention, demand-side intervention, rent/price control, anti-speculation, and multiple policies [43]. China’s 2016 tightening policy was a kind of anti-speculation policy that attempted to prevent super-exponential growth in housing prices. In this study, we argue that the soft landing following the tightening policy represented an ideal result, driving away short-term speculative investors and avoiding a crash. However, future studies may reconsider the effectiveness of China’s tightening policy by examining housing affordability.

Finally, we consider social housing, which is another significant aspect of the property market and housing policy. In transitional economies, real estate properties have been privatized or restored, the social housing sector has been downsized, and the remaining social housing is concentrated among the society’s most vulnerable groups [44]. Housing problems have been moved to the private sector, and most new houses are built by commercial providers in transitional countries and developed countries in Northwestern Europe [45]. Nevertheless, social housing still has many advantages; for example, it can lead to sustainable and balanced development, does not concentrate on central urban areas, and offers houses to socially vulnerable people that suffer from the housing affordability problem. Moreover, social housing directly or indirectly affects the commercial housing market in terms of price, supply, and demand [46]. Accordingly, policymakers must also consider the social housing sector. Specifically, it is preferable to consider the social housing sector to manage different prominent issues simultaneously when devising or evaluating real estate policy. In this sense, the Chinese government should initiate future policies to prevent a housing price crash and reconcile social problems for general social welfare.

## 5. Conclusion

The Chinese housing market has experienced an unprecedented boom since 2008. Some analysts have expressed concerns, posing dire warnings of the imminent collapse of the Chinese housing market. Others have rejected the bubble hypothesis, justifying the booming real estate market in China by referencing the rapid pace of urbanization and other social factors such as the cultural urge to invest in real estate [47]. In this context, we employ LPPL to determine the present status of China’s real estate market and how the Chinese housing market was shaped by the 2016 tightening policy. The findings demonstrate that the policy has served as an efficient means to drive away short-term speculative investors while preventing large-scale stampedes in real estate markets that would eventually result in a crash.

We propose an improved MPGA that can search the global space for optimal parameters more effectively than the standard GA, relaxing the upper constraint on the power law exponent, providing evidence of a bubble in the Chinese housing market before the 2016 tightening policy. However, after policy implementation, in contrast to Japan, the Chinese housing market did not crash, but landed softly. We attribute this difference in market reactions with the concept of susceptibility, referring to housing market sensitivity to global perturbations, arguing that low sensitivity reduced the possibility of a large number of agents simultaneously taking the same position and shifted the market to a soft landing state.

We conclude that when government policies are implemented with accurate timing, this will help stabilize housing prices and improve market conditions. At the same time, governments must exercise extreme caution to avoid overuse when enacting policies that shift demand; otherwise, the market could become immune to such policies as was evident for the fiscal expansion in South Korea in 2017, where system susceptibility is extremely low. However, policymakers should also consider various issues in the housing market, such as housing affordability and social housing, in addition to the possibility of a bubble crash to ensure that the housing market benefits general social welfare.

## Supporting information

S1 Appendix Multiple population genetic algorithm. (DOCX)

S1 Data (XLSX)

## Abbreviations

ADF: Augmented Dickey–Fuller
CAR: Capital adequacy ratio
CESEE: Central, Eastern, and Southeastern Europe
GA: Genetic algorithm
LPPL: Log-periodic power law
LTV: Loan-to-value
MPGA: Multiple population genetic algorithm
MRR: Marginal reserve requirement
OU: Ornstein–Uhlenbeck
PP: Phillips–Perron
RMSE: Root mean square error
