# Supplementary material for: Tightening policy and housing price bubbles: Examining an episode in the Chinese housing market
Source: PLoS One. 2024 Sep 6;19(9):e0309483. doi: 10.1371/journal.pone.0309483 (PMC11379212; doi:10.1371/journal.pone.0309483)
Supplement: S1 Appendix — (DOCX) [file pone.0309483.s001.docx]

**S1 Appendix. Multiple population genetic algorithm.**

The multiple population genetic algorithm (MPGA) differs from traditional genetic algorithms (GAs) in searching for optimal parameters (particularly the nonlinear parameters in the LPPL model) as it works on multiple populations evaluating each subinterval globally. If the optimization criteria are not met after the initial populations are generated, new populations are generated and the search resumes. This process continues until the optimization criteria are met. The first step in the MPGA is to generate multiple populations. Inspired by the biological concepts of crossover and mutation, each population in the MPGA can be muted into hundreds of chromosomes in which each chromosome represents a feasible solution for the four nonlinear parameters in the LPPL model. The MPGA computes the residual sum of squares (RSS) between the historical price at time t and the results from the LPPL model to determine each chromosome’s (i.e., the four nonlinear parameters) goodness of fit generated from all populations. The framework is summarized briefly as follows:

Step 1: If it is the first cycle of optimization, randomly generate the initial individuals; otherwise, choose the initial individuals from the previous cycle.

Step 2: Generate multiple populations with different characteristics. We generate 10 populations using a two-point crossover for five populations and uniform crossover for the other five.

Step 3: Each population evolves once. The individual with the highest fitness for each population migrates to the next population to replace the individual with the lowest fitness. Among these 10 best individuals, the one with the highest fitness is called the “elite.” After each evolution, the elite is recorded in the elite pool.

Step 4: If the elite no longer changes (10 or 20 generations), this is considered to be the end of one fitting. Then return to Step 1, using this elite as the initial value of the next fitting.

Step 5: If the minimum fitness value of all populations does not change, this elite represents the global minimum; otherwise, we obtain a new elite and return to Step 1.^[[1]](#footnote-1),^^[[2]](#footnote-2)^

The crossover operator is the most important element for maintaining population diversity among the four self-defined operators in GAs—ranking, selection, crossover, and mutation. The two-point crossover has a greater chromosome crossing range than uniform crossover, and performs better on global search, whereas uniform crossover is better suited for fine-tuning. As a result, multiple populations work better than any single population when combining such heterogeneous populations. Figure A1 illustrates single-point, two-point, and uniform crossover processes, while the flow chart of the entire process is presented in Figure A2.


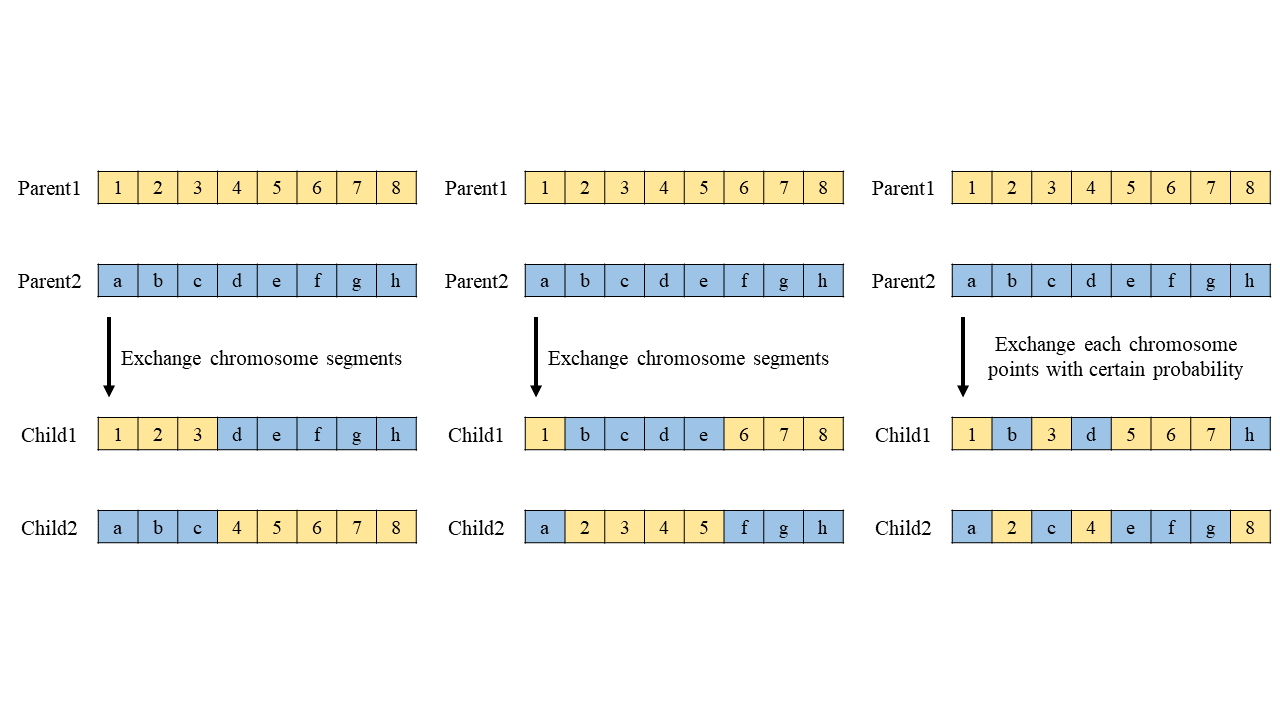


Figure A1: Illustration of single-point, two-point, and uniform crossover from left to right.


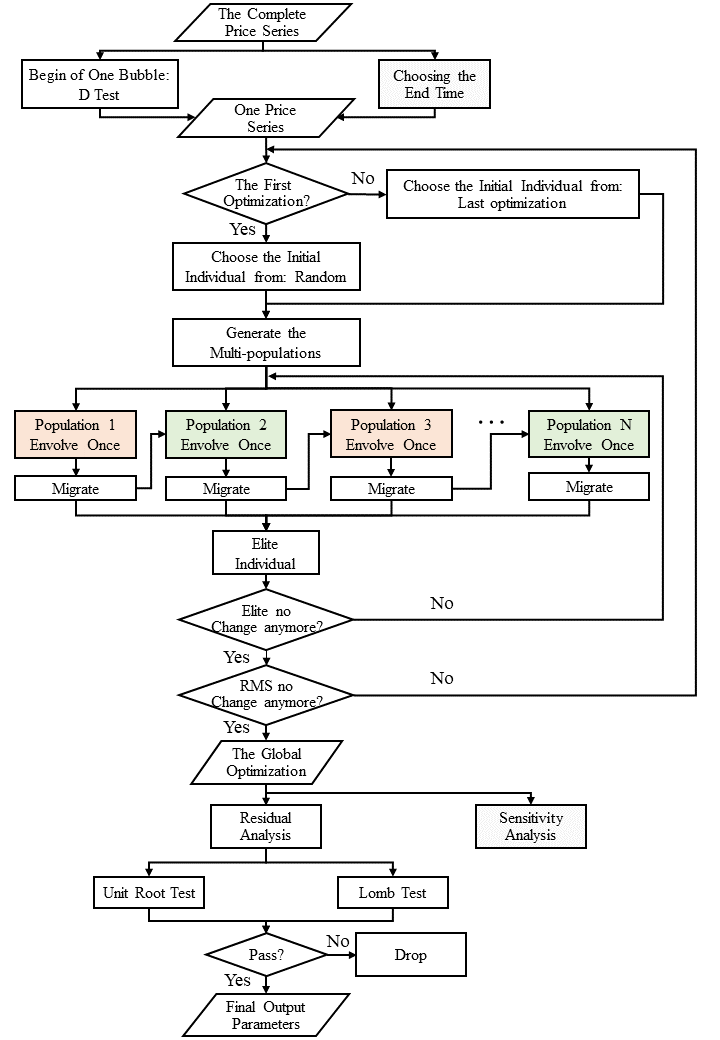


Figure A2: Multiple population genetic algorithm.

1. We convert RSS into the root mean squared errors (RMSE) to determine whether the difference between the two most recent RMSEs is less than 0.01%. [↑](#footnote-ref-1)
2. We assume staggering in our framework, allowing different populations with different crossovers to select the best individual through migration. [↑](#footnote-ref-2)
